# SchizoGoogLeNet: The GoogLeNet-Based Deep Feature Extraction Design for Automatic Detection of Schizophrenia

**DOI:** 10.1155/2022/1992596

**Published:** 2022-09-08

**Authors:** Siuly Siuly, Yan Li, Peng Wen, Omer Faruk Alcin

**Affiliations:** ^1^Institute for Sustainable Industries & Liveable Cities, Victoria University, Melbourne, Australia; ^2^School of Mathematics, Physics and Computing, University of Southern Queensland, Toowoomba, Australia; ^3^School of Engineering, University of Southern Queensland, Toowoomba, Australia; ^4^Department of Electrical and Electronics Engineering, Turgut Ozal University, Malatya, Turkey

## Abstract

Schizophrenia (SZ) is a severe and prolonged disorder of the human brain where people interpret reality in an abnormal way. Traditional methods of SZ detection are based on handcrafted feature extraction methods (manual process), which are tedious and unsophisticated, and also limited in their ability to balance efficiency and accuracy. To solve this issue, this study designed a deep learning-based feature extraction scheme involving the GoogLeNet model called “SchizoGoogLeNet” that can efficiently and automatically distinguish schizophrenic patients from healthy control (HC) subjects using electroencephalogram (EEG) signals with improved performance. The proposed framework involves multiple stages of EEG data processing. First, this study employs the average filtering method to remove noise and artifacts from the raw EEG signals to improve the signal-to-noise ratio. After that, a GoogLeNet model is designed to discover significant hidden features from denoised signals to identify schizophrenic patients from HC subjects. Finally, the obtained deep feature set is evaluated by the GoogleNet classifier and also some renowned machine learning classifiers to find a sustainable classification method for the obtained deep feature set. Experimental results show that the proposed deep feature extraction model with a support vector machine performs the best, producing a 99.02% correct classification rate for SZ, with an overall accuracy of 98.84%. Furthermore, our proposed model outperforms other existing methods. The proposed design is able to accurately discriminate SZ from HC, and it will be useful for developing a diagnostic tool for SZ detection.

## 1. Introduction

Schizophrenia (SZ) is a devastating mental disorder and a progressive neurological disease that causes a significant impact on the quality of life of patients and their families, social environments, and healthcare systems [1]. This disorder affects a person's perception of reality, social interactions, thought processes, and cognitive ability. Symptoms of SZ include hallucinations (hearing voices or seeing things that are not there), delusions (fixed, false beliefs), and thought disorders (unusual ways of thinking), as well as reduced expressions of emotions, reduced motivations to accomplish goals, difficulty in social relationships, motor impairments, and cognitive impairments [2, 3]. SZ affects every 1 in 100 Australians and approximately 21 million people worldwide [4, 5]. It is associated with increased morbidity and mortality, and is the cause of disability and health costs worldwide [6]. SZ is treatable, but its treatment involves long-term medications, causing an extreme burden on healthcare systems and families. If the patients are not treated, SZ symptoms may be persistent which makes them disabled after a period of time. If early detection is possible, then patients can get timely treatments that can help those affected individuals and their families improve their lives [7]. Hence, there is a growing demand to develop an efficient and automatic diagnostic technique for the early detection of SZ patients from healthy control (HC) people.

Traditionally, the diagnosis of SZ is mainly performed through solely interviews and observations of patient behavior by a trained psychiatrist [8]. This diagnosis is a manual process that is time-consuming, burdensome, and subject to human errors and bias. Instead of this, recently, some imaging techniques, such as magnetic resonance imaging, computed tomography, positron emission tomography, and electroencephalography (EEG) have been introduced to diagnose SZ. Among these imaging techniques, currently, EEG has emerged as the reference standard for diagnosing SZ due to its high temporal resolution, noninvasiveness, and relatively low financial cost compared to other tests [2, 9]. EEG signals describe the electrical activity of the human brain recorded from the scalp via a set of electrodes. EEG recording generates a huge volume of data. Generally, this massive amount of data is analysed by visual inspection, which is time-consuming, error-prone, and reduces decision-making reliability. Thus, a computer-aided automatic data analysis system is required to make an accurate and reliable decision for the diagnosis of SZ.

### 1.1. Related Works

In recent years, many researchers have attempted to identify SZ from EEG data [10–18], but no efficient and reliable system has been developed yet for the reliable detection of SZ patients. Much research has been performed based on traditional handcrafted feature extraction methods, but they were inadequate in their ability to generate suitable performance for real-time applications. For example, Sabeti et al. [10] used autoregressive (AR) model coefficients, band power, and fractal dimension-based features for identifying SZ subjects. These features were fed to linear discriminant analysis (LDA), multiLDA, and adaptive boosting (AdaBoost) classifiers. An empirical mode decomposition (EMD) technique was introduced in [2] for the diagnosis of SZ from EEG signals to handle the behavior of nonstationary and nonlinear EEG signals. In [11], Ramos et al. employed power spectral density-based features in the maximum likelihood theory for classifying SZ and HC subjects.

Kaplan et al. [12] used spectral features, and the obtained features were classified using the “Kora-N” algorithm. A fast Fourier transformation (FFT)-based feature extraction process was reported by Buettner et al. in [13]. Finally, those features were used as input to a random forest (RF) classification method for identifying SZ and non-SZ. Approximate entropy, Shannon entropy, Kolmogorov complexity, and Lempel-Ziv complexity methods were proposed by Akar et al. in [14] for extracting features from EEG signals for identifying SZ. In their another work, Akar et al. [15] computed features using wavelet transformation (WT) and Welch power spectral density (PSD) methods for the detection of schizophrenia from EEG data. In [16], Li et al. used functional EEG networks to extract the inherent spatial pattern of the network (SPN) feature for brain states. The combined SPN features of the rest and task networks were used as the input to LDA and the support vector machine (SVM) to recognize SZ.

Only a few studies have been performed on deep learning for the detection of SZ using EEG data. In deep learning, both the feature extraction and classification processes are conducted automatically, while traditional techniques require features to be extracted manually. Phang et al. [17] proposed a deep convolutional neural network (CNN) framework for the classification of SZ. In that model, the authors integrated the features from various domains and dimensions using different fusion strategies, and the model achieved 93.06% accuracy. An eleven-layered CNN model was introduced by Oh et al. in [18] to analyse the EEG signals for the diagnosis of schizophrenia. The model generated a classification accuracy of 81.26% for subject-based testing.

### 1.2. Motivations

Most of the existing research studies for SZ detection employ handcrafted feature extraction methods (e.g., WT, FFT, EMD, PSD, and entropy) before classification [10–16]. These feature extraction methods are manually chosen based on researchers' expertise. The obtained performances of those methods are not satisfactory. The handcrafted feature extraction methods cannot form intellectual high levels of representations of EEG signals to discover deep concealed characteristics from data that can achieve better performance. This manual feature extraction process is time-consuming, labor-intensive, and has bias. Furthermore, if sizes of data are large, the method may not run properly and sometimes underperform.

As mentioned before, very few studies used deep learning methods for the detection of schizophrenia from EEG [17, 18]. The significant characteristic of deep learning is that the model can automatically extract effective features, which has certain advantages for large-scale data. The developed deep learning-based SZ detection methods are still limited in their ability to balance efficiency and accuracy. Hence, this study is motivated to introduce a new deep learning-based feature extraction scheme for automatic and efficient identification of SZ patients using EEG.

### 1.3. Objectives of This Study

The key objective of this study is to introduce a deep learning-based feature extraction scheme involving the GoogLeNet model called “SchizoGoogLeNet” for automatically and efficiently distinguishing schizophrenic patients from HC subjects using EEG data with improved performance. The reason for considering the GoogLeNet model in this study is that the GoogLeNet architecture has the ability to produce better performance than other deep learning models as it is designed to be a powerhouse with increased computational efficiency. This model trains faster than other network methods (e.g., AlexNet and VGGNet) and has fewer parameters and lower computational complexity than other models [19, 20]. Also, it has a relatively lower error rate than other deep learning methods.

To our knowledge, for the first time, GoogLeNet was introduced in SZ detection using EEG signals in this study. Our proposed “SchizoGoogLeNet” framework consists of several steps. First, this study employs an average filtering method to remove noise and artifacts from the raw EEG signals. Second, significant hidden features are extracted from EEG signals to identify schizophrenia patients from HC subjects. Finally, a sustainable classification method for the obtained deep GoogLeNet features is explored. At this stage, deep learning and machine learning methods are tested for the obtained deep feature set. The performance of the proposed framework was tested using a publicly available EEG dataset.

### 1.4. Our Contributions

The main contributions of this work are summarized as follows: (1) For the first time, we introduce a GoogLeNet-based deep feature extraction scheme for automatic detection of SZ using EEG signals; (2) we investigate the impact of balance and unbalanced datasets on the proposed SZ detection model; (3) we discover a sustainable classifier for the obtained GoogLeNet deep feature set in a deep learning and machine learning environment; (4) we explore the performances of the deep feature set with a GoogLeNet classifier and several machine learning methods; (5) we achieve improved performances for our proposed model compared to those of the existing methods.

The rest of this paper is arranged as follows: The proposed methodology is described in Section 2. This section also provides the description of the data that is used in this study.  Section 3 provides the experimental setup and the results with their corresponding discussions, followed by the conclusion in Section 4.

## 2. Proposed Methodology

**Figure 1:**
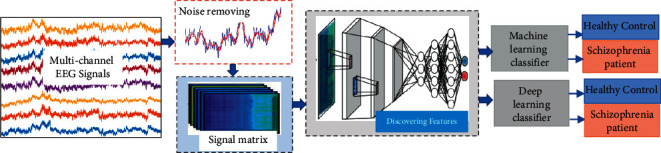
The overall architecture of the proposed “SchizoGoogLeNet” framework for automatic identification of SZ from EEG signals.

This study proposes a GoogLeNet-based deep feature extraction scheme, “SchizoGoogLeNet,” for detecting SZ from EEG data automatically.  Figure 1 graphically presents the general architecture of the proposed strategy. The proposed scheme involves four stages, such as data acquisition, removing noise and artifacts using average filtering, discovering deep features using GoogLeNet, and exploring sustainable classifiers for the obtained features. A detailed description of these steps is provided below.

### 2.1. Data Acquisition

This study used EEG data collected from 81 subjects, including 49 schizophrenia patients and 32 normal control persons, from the Kaggle data source. These data consist of EEG recordings of 14 female and 67 male subjects. The average age is 39 years, and the average education level is 14.5 years. The EEG data were recorded from 64 scalp sites and 8 external sites using a BioSemi active two system (http://www.biosemi.com). The data were continuously digitized at 1024 Hz and referenced offline to averaged earlobe electrodes. The details of the dataset can be seen online at https://www.kaggle.com/broach/button-tone-sz [21]. The description of the data is also available in [22].

### 2.2. Removing Noise and Artifacts Using Average Filtering

This section aims to introduce a technique for reducing noise and artifacts of EEG signals and improving the signal-to-noise-ratio (SNR) as the signal data are very noisy and often affected by artifacts. Due to noise, EEG signals have a low SNR that may lead to incorrect conclusions. The noise-removing processes improve the data quality to create an appropriate model. In this study, we employ an average filter (AF) technique for removing noise and artifacts from EEG signals [23]. The reason for using this filtering is that it is simple, intuitive, and easy to implement for smoothing the signals and for dropping the amount of intensity variations that can reduce undesirable information from the signals.

**Figure 2:**
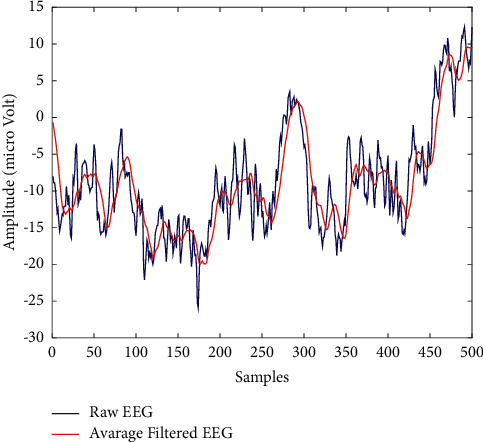
An illustration of the pattern of a raw EEG and filtered EEG signal.

In this study, we reduced noise smoothing of each signal (*Sg*) by the AF technique with a kernel size of KS = 12, and then, we sampled the smoothed signal considering an interval length IL = KS = 12 which is denoted as $\hat{\text{Sg}}$. We selected the values of KS and IL using a trial and error procedure to make the final matrix with a size of around 200. After the filtering, the raw signal data become the input matrix in the feature extraction stage of the GoogLeNet model (discussed in the next step).  Figure 2 shows an example of the pattern of a raw EEG signal and a filtered EEG signal. It is apparent from Figure 2 that the shape of the filtered signal is the same as that of the raw signal. As can be seen in Figure 2, the number of data points has been reduced while still keeping the shape of the original curve of the EEG signal.

(1) $$\begin{array}{c} \bar{\text{Sg}}\left(\text{t}\right)=\frac{1}{KS}\overset{t+KS/2}{\underset{i=t-KS/2}{\sum }}Sg\left(i\right). \end{array}$$

(2) $$\begin{array}{c} \hat{\text{Sg}}\left(\text{t}\right)=\overset{L}{\underset{i=0}{\sum }}\delta \left(i-t\cdot IL\right)\cdot \bar{\text{Sg}}\left(i\right). \end{array}$$

### 2.3. Discovering Deep Features Using GoogLeNet

Technically, a feature represents a distinguishing property, a recognizable measurement, and a functional component obtained from a segment of a pattern [24]. Extracted features convey the most important information for the classification stage. Sometimes, traditional handcrafted features cannot convey meaningful information about the SZ detection due to manual choice of methods and also cannot handle big sizes of data. This section's goal is to discover the significant feature set from EEG signals using the deep learning method that empowers efficiently the recognition of SZ from HC subjects. For this purpose, this study introduces the deep GoogLeNet-based architecture to extract representative features for identifying SZ from the denoised EEG signals automatically. To our knowledge, for the first time, GoogLeNet is employed in this study for the detection of SZ from EEG signals. GoogLeNet is a convolutional neural network (CNN)-based architecture designed by researchers at Google. It was the winner of ImageNet 2014, where it proved to be a powerful model.

**Figure 3:**
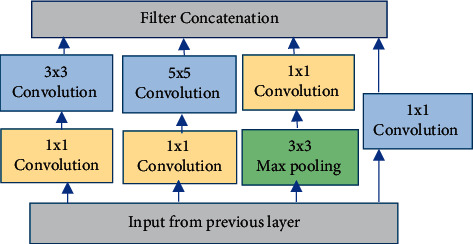
General architecture of the inception block.

The main objective of the GoogLeNet architecture is to achieve high accuracy with a reduced computational cost [19, 20]. The GoogLeNet model is constructed based on the inception architecture that introduced the new concept of the inception block in CNN, whereby it incorporates multiscale convolutional transformations using split, transform, and merge ideas [20]. A general strategy of the inception block is illustrated in Figure 3. The inception module is different from other deep learning architectures where there is a fixed convolution size for each layer. In the inception module, 1 × 1, 3 × 3, and 5 × 5 convolutions and 3 × 3 max pooling perform in a parallel way at the input, and the output of these is stacked together to generate the final output. In the GoogLeNet model, conventional convolutional layers are replaced with small blocks. These blocks condense filters of different sizes (e.g., 1 × 1, 3 × 3, and 5 × 5) to capture spatial information at different scales, including both fine and coarse grain levels [19, 20]. As shown in Figure 3, multiple convolutions, with 1 × 1 filters, 3 × 3 filters, and 5 × 5 filters, and 3 × 3 max-pooling layers are organised in the GoogLeNet model.

The GoogLeNet model regulates the computations by adding a bottleneck layer of 1 × 1 convolutional filters before employing large-size kernels. 1 × 1 convolution is used to decrease the number of parameters (weights and biases) of the architecture. Furthermore, it uses sparse connections (not all the output feature maps are connected to all the input feature maps) to overcome the problem of redundant information and reduced costs by omitting feature maps that are not relevant [20]. Additionally, connection density is reduced by using global average pooling at the last layer instead of using a fully connected layer. These parameter tunings cause a significant decrease in the number of parameters [25].

In this study, we designed the structure of the GoogLeNet model for implementation of the SZ EEG database as illustrated in Table 1. This table presents layer-by-layer architectural details of the GoogLeNet model. “#1 × 1 #3 × 3 #5 × 5” refers to various convolution filters used within the inception module. “#3 × 3 reduce” and “#5 × 5 reduce” symbolize the number of 1 × 1 filters in the reduction layer used before related convolution layers. The number of 1 × 1 filters in the projection layer after the built-in maximum pooling is shown in the “pool projection” column (denoted as “Pool proj”). “Max pool” stands for the maximum number of pooling layers. The purpose of these max-pooling layers is to downsample the input as it is fed forward through the network. All the convolution, reduction, and projection layers inside this architecture use rectified linear units (ReLUs) as their activation functions. This architecture is 22 layers deep without pooling (or 27 layers when we count pooling) [19, 20].

### 2.4. Exploring Sustainable Classification Method for Identifying Schizophrenia

This section's aim is to discover a sustainable classifier for the obtained deep feature set to classify SZ and HC subjects and improve its performance. Unlike other deep learning models, GoogLeNet does not use fully connected (fc) layers for the final result of classification. Instead, the last convolutional map is subjected to channel-wise global average pooling, and the average activation values of each of the channels are used as the feature vector of the input image.

As can be seen in Table 1, the last four layers of our proposed architecture are as follows: a dropout layer set with a probability of 40% dropout, a fully connected (fc) layer, a softmax layer, and a classification output layer. The softmax layer is a final layer of the model that uses the softmax function, and an activation function is used to derive the probability distribution of a set of numbers within an input vector. The output of a softmax activation function is a vector in which its set of values represents the probability of a class or an event occurrence. The classification output layer is set to have the same size as the number of classes in the new dataset, which was two (e.g., SZ and HC) in our case. Before training GoogLeNet, training parameters were set after empirical evaluation. In our experiments, two parameters were used to access the performance of the networks: maximum epochs and batch size.

To determine an appropriate classifier for the obtained deep GoogLeNet feature set, this study also tested four machine learning classification methods: SVM, k-nearest neighbour (KNN), decision tree (DT), and linear discriminant analysis (LDA) for identifying SZ from HC subjects. The reason for the choice of these classifiers in this study is due to their popularity, simplicity, and effectiveness in implementation. They are also very powerful and fast learning algorithms that examine all their training inputs for classification. The description of these methods is available in [26–30].

### 2.5. Performance Evaluation Parameters

To fairly assess the performance of the proposed models, we computed all standard measurement parameters including the accuracy, sensitivity, specificity, positive predictive value, false alarm rate, *F*1-score, and the receiver operating characteristic curve (ROC) in this study. The descriptions of the mentioned measurements are available in [28, 31–35].

## 3. Experiments and Results

### 3.1. Experimental Setting

In this study, we performed all the experiments in MATLAB (2018b) on a PC with a six-core Intel i7 processor and 32 GB of memory. The server was equipped with an NVIDIA RTX 2060 GPU with 6 GB of memory. We run the GoogLeNet model in the MATLAB deep learning toolbox for our proposed design. As stated before, the dataset includes a total of 81 subjects, where 32 cases are normal control (with 3108 trials; 3072 samples per trial; 70 channels) and 49 cases are schizophrenia patients (with 4608 trials; 3072 samples per trial; 70 channels). In this study, we used all 70 channels' data for the proposed design. Here, we provide an example how we processed the raw EEG signal data of each subject and transformed the processed dataset for implementation in the experiments in this study. For example, for Subject 1, we had a dataset: 887808 × 70 (samples x channels), and we converted this dataset to a matrix sized 70 × 3072 × 289 (channels x window length × epoch) using the transpose process. Here, we obtained epoch = 289 dividing samples by window length (887808/3072). After the average filtering, the raw signal data matrix 70 × 3072 × 289 was moved to a reduced matrix size of 70 × 256 × 289. Afterward, the 70 × 256 × 289 matrix was resized to 224 × 224 × 3 × 289 to be compatible with the deep GoogLeNet input size for subject 1.

Following a similar process, for a total of 81 subjects, the whole dataset was transformed into an image matrix with a size of 224 × 224 × 3 × 23201 (height × weight × 3 symbolize color layer × image samples). The sizes of data for SZ and HC are 224 × 224 × 3 × 13975 and 224 × 224 × 3 × 9226, respectively, which shows that the sample points of SZ and the HC groups are not equal. Thus, we divided the dataset into two groups: balanced and unbalanced datasets to test the effect of equal and unequal sizes of sample points in SZ and HC categories. A balanced dataset is one that has the same number of observations for each class in a classification dataset. An unbalanced dataset has the different number of observations for each class. Both SZ and HC categories in this study's balanced dataset have the same number of sample points; however, both categories in the unbalanced dataset have an unequal number of sample points. As seen in Table 2, the balanced dataset consists of 9226 sample points in each category of SZ and HC (including training, validation, and testing data), and total sample points for both categories are 18,452.

In the unbalanced dataset, the SZ category has 13,975 sample points and the HC category has 9,226 sample points (including training, validation, and testing data). The total sample point size for the unbalanced data is 23,201. Please note that the unbalanced dataset is the original dataset after data preprocessing. Thus, the balanced data size is 224 × 224 × 3 × 18452 (height x weight x 3 symbolize color layer x image samples), and the unbalanced data size is 224 × 224 × 3 × 23201 (height × weight × 3 symbolize color layer × image samples). Then, both datasets are divided into three parts: training, validation, and testing with a ratio of 70%, 10%, and 20%, respectively. The sizes of different parts of data are given in Table 2. In this study, the training dataset was used for the learning process in the proposed model, and the validation dataset was regarded as a part of the training set to tune the model. The validation set was used for tuning the parameters of the model and also for avoiding overfitting. Generally, the validation dataset helps provide an unbiased evaluation of the model's fitness. The testing dataset was used for the performance evaluation.

### 3.2. Feature Extraction Process and Hyperparameter Setting

**Figure 4:**
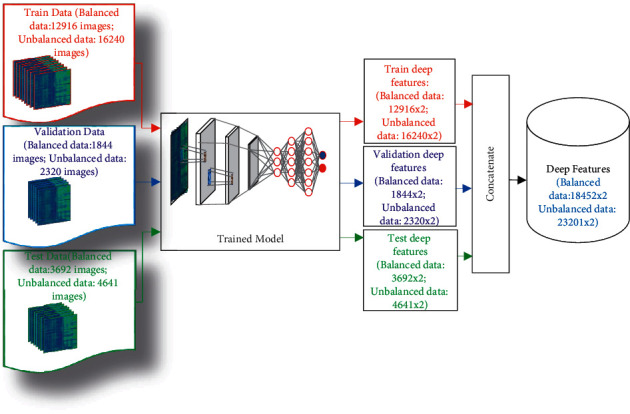
Feature extraction process in the proposed “SchizoGoogLeNet” model for the balanced and unbalanced dataset.

This section presents the process of how the features are extracted using the GoogLeNet model for the balanced and unbalanced datasets.  Figure 4 shows the feature extraction process in the GoogLeNet model for the balanced dataset and unbalanced dataset. As seen in Figure 4, the proposed GoogLeNet model yields a deep feature set with a size of 18452 × 2 for the balanced dataset and 23201 × 2 for the unbalanced dataset. It means that two deep features are generated, including 18,452 sample points for the balanced dataset and 23,201 for the unbalanced dataset. In both datasets, two deep features are called deep feature1 and deep feature 2.

**Figure 5:**
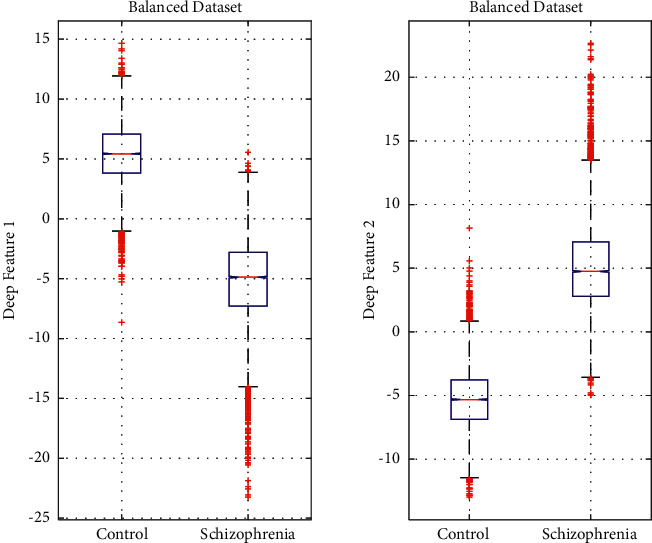
Distribution of two deep features for the balanced dataset.

**Figure 6:**
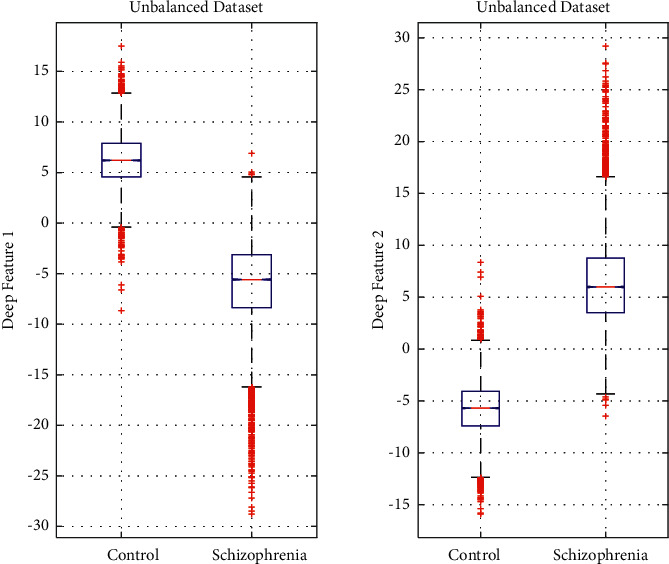
Distribution of two deep features for the unbalanced dataset.

Figures 5 and 6 present the patterns of the distribution of the obtained two deep features (deep feature 1 and deep feature 2) for the balanced and unbalanced datasets through boxplots, respectively. As can be seen in both figures, the shape of the distribution in both schizophrenia and control groups are symmetrical and there are some outliers in each diagram. In both figures, it is observed that there is a significant difference between the central value of schizophrenia and control groups in both feature sets. The boxplot figures clearly demonstrate that there is a clear and significant difference in the values of the feature set in two groups that help in the efficient classification of schizophrenia and control.

To find the best model, the hyperparameters (e.g., the number of hidden units, the number of epochs, and the batch size) of the GoogLeNet model are optimized (tuned) by the training process. We run the data through the operations of the model, compare the resulting prediction with the actual value for each data instance, evaluate the accuracy, and adjust until we find the best values. We performed an extensive number of experiments to find appropriate values for different parameters. The configuration of hyperparameters of the proposed model is provided in Table 1. The table illustrates the layer-by-layer structural details of the proposed “SchizoGoogLeNet” model.

In this study, the SVM classifier with a linear kernel was used as an optimal kernel function after testing all the kernels (e.g., linear, polynomial, and radial basis kernels) because this kernel produced a better performance compared to others. The KNN classifier used the default distance metric “Euclidean distance” for the distance measure and *K* = 1 in the model after several experimental evaluations. For DT and LDA classifiers, the parameter values are considered which have been used in MATLAB default parameter settings as there are no specific guidelines for setting the values of the parameters for these classifiers.

### 3.3. Results and Discussion

This section provides the experimental results that are achieved using 10-fold cross-validation through MATLAB. For both balanced and unbalanced datasets, we used the obtained deep feature set as an input to the softmax classifier of GoogLeNet (deep learning (DL) classifier) and also four popular machine learning (ML) classifiers (SVM, KNN, DT, and LDA), separately, to find an optimal classifier. Tables 3 and 4 present the overall performance results for our proposed approaches in terms of the sensitivity (SEN), specificity (SPE), accuracy (ACC), positive predictive value (PPV), and *F*1-score for the balanced and unbalanced datasets, respectively.

As can be seen in Table 3, for the balanced dataset, the SVM classifier achieves the highest performances such as ACC (98.30%), SPE (98.27%), PPV (98.27%), and *F*1-score (98.30%), and the LDA classifier produces the highest sensitivity value (98.53%). The lowest performances (e.g., ACC (94.28%), SEN (92.15%), SPC (96.42%), PPV (96.26%), and *F*1-score (94.16%)) are obtained by the GoogLeNet classifier (DL classifier). For the unbalanced dataset, Table 4 reports that among the reported classifiers, the highest classification performances are attained by the SVM classifiers, which are 98.84% of ACC, 99.02% of SEN, 98.58% of SPE, 99.06% of PPV, and 99.04% of *F*1-score. On the other hand, the lowest performance (e.g., ACC (95.09%), SEN (93.81%), SPC (97.02%), (PPV 97.95%), and *F*1-score (95.83%)) are obtained by the GoogLeNet classifier like the balanced dataset.

**Figure 7:**
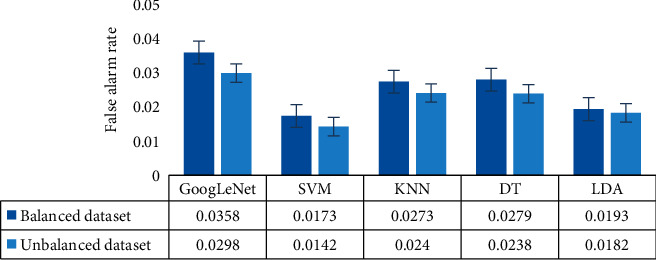
False alarm rate (FAR) for all of the reported classifiers for the balanced and unbalanced datasets.

In order to further assess, we also computed the false alarm rate (FAR) of the proposed classification models for the balanced and unbalanced datasets as shown in Figure 7. This figure also demonstrates that the SVM classifier produces better performance (a lower FAR indicates better performance) with the obtained feature set than the GoogLeNet classifier (a higher FAR indicates lower performance). From Tables 3 and 4 and Figure 7, it is clearly apparent that the obtained feature set yields higher performance with ML classifiers than the DL classifier (e.g., GoogLeNet) for both balanced and unbalanced datasets. In both datasets, the SVM classifier is superior for the obtained deep feature set compared to other reported classifiers, and the GoogLeNet classifier with the same feature set achieved the worst performance.

**Figure 8:**
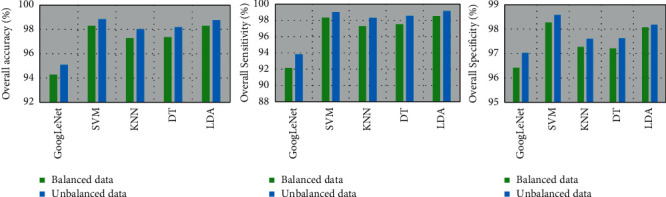
Comparison of performances between the balanced and the unbalanced datasets.

Figure 8 and also Figure 7 show a comparison of the performances (in terms of ACC, SEN, SPE, and FAR) between the balanced and unbalanced datasets. The figures demonstrate that the performances of all classifiers are higher for the unbalanced dataset than those for the balanced dataset. The overall accuracy is increased by 3.75% for the unbalanced data and 4.02% for the balanced data for the ML-based classifier compared to the DL scheme. The unbalanced dataset's improved performance may be due to the fact that it is the original dataset, which includes all of the subjects' data points. On the other hand, the balanced dataset was produced from the unbalanced dataset by eliminating some subjects. The performance was lower for the balanced dataset because some subjects' data points had been eliminated. From the results, it can be considered that the obtained deep feature set with an SVM classifier is exceptional for the identification of SZ EEG signals from HC.

**Figure 9:**
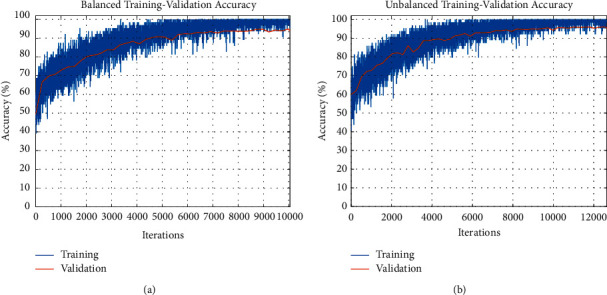
(a) Patterns of the training and validation accuracy of the GoogLeNet-based model for the balanced dataset. (b). Patterns of the training and validation accuracy of GoogLeNet-based model for the unbalanced dataset.

Figures 9(a) and 9(b) display an illustration of training and validation accuracy patterns for the deep GoogLeNet model in different iterations of the balanced and unbalanced datasets, respectively. For both datasets, the training accuracy and validation accuracy increase with the increase in the iteration numbers. It is notable that the accuracy of the training set does not deviate substantially from that of the validation set as observed in Figures 9(a) and 9(b). In the training stage, the learning rate was set at 0.0001 and the batch number was one sample each time. The number of filters and kernel size was determined via the brute force technique.

**Figure 10:**
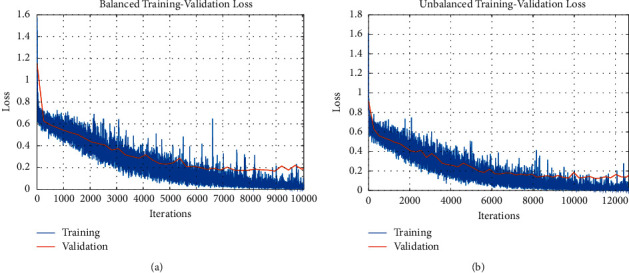
(a) Patterns of the training and validation loss information of the GoogLeNet-based model for the balanced dataset. (b). Patterns of the training and validation loss information of the GoogLeNet-based model for the unbalanced dataset.

The loss information of the training set and validation set with respect to different iterations is displayed in Figures 10(a) and 10(b) for the balanced dataset and unbalanced dataset, respectively. For both datasets, it is observed that the training loss and the validation loss decrease with the increase in the iteration numbers. The performance of the training set does not significantly diverge from that of the validation set, as shown in Figures 10(a) and 10(b).

**Figure 11:**
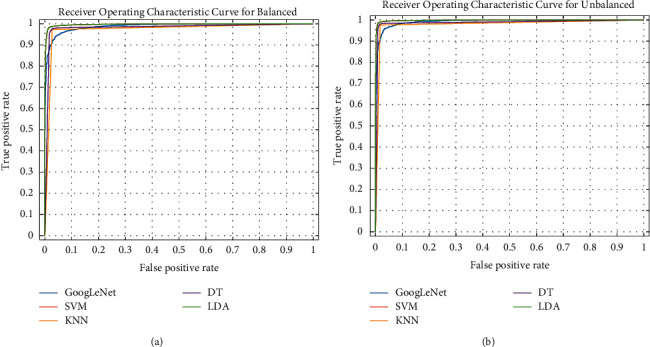
(a) The ROC curve for different classification models with the obtained deep feature set for the balanced dataset. (b) The ROC curve for different classification models with the obtained deep feature set for the unbalanced dataset.

To further assess the effectiveness of the GoogLeNet-based model, the ROC curves are drawn for different SZ detection models, where the input data were the deep feature set shown in Figures 11(a) and 11(b) for the balanced and unbalanced datasets, respectively. The corresponding performance measurements in every condition are shown in Table 5. Table 5 reports the area values under the ROC curve (AUC) for the reported classifiers. The AUC is the value of the area under the ROC curve that belongs to a value between 0 and 1 (a larger area indicates a better performance of the classifier). As can be seen in Table 5, the highest AUC is obtained by the SVM classifier, which is 0.9984 (close to 1) for the unbalanced dataset and 0.9973 for the balanced dataset. The KNN model produces the lowest AUC for both the balanced (0.9728) and the unbalanced (0.9795) datasets. Like the previous results, the results also indicate that the SVM classifier with the obtained deep feature set works better than other reported classifiers.

### 3.4. Comparative Analysis Report for Our Proposed Method with Existing State-of-the-Art Methods

A comparison of the prior EEG-based techniques used for SZ detection with our proposed model has been provided in Table 6. Until now, we found seven articles [2, 28, 35–39] in the literature for the same database that we have used in this study. Table 6 shows the performance comparison of the proposed method with these published methods [2, 28, 35–39]. Kahre et al. [35] reported a method based on empirical wavelet transformation and SVM for the detection of SZ from EEG signals. Their method achieved an ACC of 88.70%, SEN of 91.13%, and SPE of 89.29. In [2], Siuly et al. introduced empirical mode decomposition (EMD)-based features with an ensemble bagged tree (EBT) for the detection of SZ using EEG signals. The ACC, SEN, and SPE scores of their method were 89.59%, 89.76%, and 89.32%, respectively. Guo et al. [38] reported a random forest (RF)-based machine learning algorithm for identifying schizophrenia patients from healthy control subjects using EEG signal data. In the designed plan, the author considered a number of features such as gender, age, education, and event-related potential (ERP) and the combination of the features.

RF yielded an accuracy of 81.1%. Khare et al. [36] introduced an automatic approach based on flexible tunable *Q* wavelet transform (F-TQWT) and a flexible least square support vector machine (F-LSSVM) classifier for the detection of SZ from EEG signals. The authors used the “Fisher score” method for the selection of the most discriminant channels. Their proposed method generated 91.39% accuracy, 92.65% sensitivity, and 93.22% specificity. In [37], Guo et al. proposed a scheme based on convolutional neural networks (CNNs) to characterize the difference in the distributed structure of data for identifying SZ from EEG. Their method achieved an accuracy of 92%. Khare et al. [38] designed a model involving a robust variational mode decomposition (RVMD) and an optimized extreme learning machine (OELM) algorithm. The experiment results reveal that the third mode's chaotic features are responsible for generating the best performance (overall ACC of 92.30%). In [39], a time-frequency analysis-based convolutional neural network (CNN) model was proposed for identifying SZ from EEG signals by Khare and Bajaj. The authors used continuous wavelet transform, short-time Fourier transform, and smoothed pseudo Wigner–Ville distribution (SPWVD) techniques to obtain scalogram, spectrogram, and SPWVD-based time-frequency representation (TFR) plots for SZ detection. Their method achieved an overall accuracy of 93.36% using the SPWVD-based TFR and CNN model.

It is apparent from Table 6 that our proposed model yielded the highest performance scores with an accuracy of 98.84%, sensitivity of 99.02%, and specificity of 98.58%, compared to performance scores of the other existing methods. The achieved accuracy improvement of our proposed model is 17.74% better than the accuracy score of Zhang [28] and 10.14% better than the accuracy score of Khare et al. [35]. In the end, it can be concluded based on the experimental results that deep GoogleNet features of EEG signals with an SVM classifier could serve as an applicable measurement to correctly discriminate between schizophrenics and HC subjects. [40].

## 4. Concluding Remarks

In this study, a GoogLeNet-based feature extraction scheme, called “SchizoGoogLeNet” is developed to efficiently identify SZ patients from HC subjects using EEG signal data. The proposed GoogLeNet model automatically extracted important hidden features which are advantageous for large-scale data. The obtained deep feature set was verified by the GoogLeNet classifier (DL classifier) and also four popular ML classifiers (e.g., SVM, KNN, DT, and LDA), separately. The performance of the proposed framework was evaluated on the benchmark SZ EEG database from Kaggle through extensive experimental evaluation. To check the effect of equal and unequal sample points in SZ and HC groups, we divided the dataset into two groups: balanced (the same number of sample points in SZ and HC) and unbalanced dataset (unequal sample points in SZ and HC) (original dataset). The experimental results show that the unbalanced set produces better performance compared to the balanced dataset. Among the reported classifiers, the SVM classifier with the obtained deep feature set yielded the highest performance (e.g., ACC 98.84%, SEN 99.02%, SPE 98.58%, PPV 99.06%, and F1-score 99.04%), while the lowest performances were obtained by the GoogLeNet classifier (e.g., ACC 95.09%, SEN 93.81%, SPC 97.02%, PPV 97.95%, and F1-score 95.83%). Moreover, our proposed model outperforms the existing methods. The findings of this study indicate that the obtained deep GoogLeNet features perform better with the SVM classifier in the SZ detection than the DL classifier (GoogLeNet classifier).

This study has some limitations, such as the fact that the study only considers two-class classification problems (SZ versus HC), while we intend to expand the application of the suggested approach to multiclass scenarios soon. Another flaw is that this study used a small SZ-based EEG dataset (81 total subjects: 49 schizophrenia (SZ) patients and 32 healthy control (HC) people). In the near future, we will broaden our method's application to include huge clinical datasets.

## Figures and Tables

**Table 1:** Model architecture of GoogLeNet used in this study.

Layer	Patch size/stride	Depth	#1 × 1	#3 × 3 reduce	#3 × 3	#5 × 5 reduce	#5 × 5	Pool proj	Output size
Conv1	7 × 7/2	1							112 × 112 × 64
Max pool1	3 × 3/2	0							56 × 56 × 64
Conv2	3 × 3/1	2		64	192				56 × 56 × 192
Max pool2	3 × 3/2	0							28 × 28 × 192
Inception-3a		2	64	96	128	16	32	32	28 × 28 × 256
Inception-3b		2	128	128	192	32	96	64	28 × 28 × 480
Max pool3	3 × 3/2	0							14 × 14 × 480
Inception-4a		2	192	96	208	16	48	64	14 × 14 × 512
Inception-4b		2	160	112	224	24	64	64	14 × 14 × 512
Inception-4c		2	128	128	256	24	64	64	14 × 14 × 512
Inception-4d		2	112	144	288	32	64	64	14 × 14 × 528
Inception-4e		2	256	160	320	32	128	128	14 × 14 × 832
Max pool4	3 × 3/2	0							7 × 7 × 832
Inception-5a		2	256	160	320	32	128	128	7 × 7 × 832
Inception-5b		2	384	192	384	48	128	128	7 × 7 × 1024
Average pool5	7 × 7/1	0							1 × 1 × 1024
Dropout (40%)		0							1 × 1 × 1024
Fc		1							1 × 1 × 2
Softmax		0							1 × 1 × 2
Classification output									1 × 1 × 2

**Table 2:** The sizes of different parts of data.

Data	Category	Training data	Validation data	Testing data
Balanced	HC	224 × 224 × 3 × 6458	224 × 224 × 3 × 922	224 × 224 × 3 × 1846
	SZ	224 × 224 × 3 × 6458	224 × 224 × 3 × 922	224 × 224 × 3 × 1846
	Total	224 × 224 × 3 × 12916	224 × 224 × 3 × 1844	224 × 224 × 3 × 3692

Unbalanced	HC	224 × 224 × 3 × 6458	224 × 224 × 3 × 922	224 × 224 × 3 × 1846
	SZ	224 × 224 × 3 × 9782	224 × 224 × 3 × 1397	224 × 224 × 3 × 2796
	Total	224 × 224 × 3 × 16240	224 × 224 × 3 × 2319	224 × 224 × 3 × 4642

SZ = schizophrenia; HC = healthy control.

**Table 3:** Overall performances of the proposed methods for the balanced dataset.

Classifier	Sensitivity (%)	Specificity (%)	Accuracy (%)	Positive predictive value (%)	*F*1-score (%)
GoogLeNet	92.15	96.42	94.28	96.26	94.16
SVM	98.33	98.27	98.30	98.27	98.30
KNN	97.29	97.27	97.28	97.27	97.28
DT	97.51	97.21	97.36	97.22	97.36
LDA	98.53	98.07	98.29	98.08	98.30

**Table 4:** Overall performances of the proposed methods for the unbalanced dataset.

Classifier	Sensitivity (%)	Specificity (%)	Accuracy (%)	Positive predictive value (%)	*F*1 score (%)
GoogLeNet	93.81	97.02	95.09	97.95	95.83
SVM	**99.02**	**98.58**	**98.84**	**99.06**	**99.04**
KNN	98.30	97.60	98.02	98.42	98.36
DT	98.55	97.62	98.18	98.43	98.49
LDA	99.13	98.18	98.75	98.80	98.97

Bold values represent highest performance.

**Table 5:** AUC values for the proposed SZ detection models.

Models	Balanced dataset	Unbalanced dataset
	Area under curve (AUC) values	Area under curve (AUC) values
GoogLeNet	0.9884	0.9923
SVM	0.9973	**0.9984**
KNN	0.9728	0.9795
DT	0.9815	0.9874
LDA	0.9973	0.9984

**Table 6:** The comparison of the proposed method with other methods for the same database.

Authors	Methods	ACC (%)	SEN (%)	SPE (%)
Khare et al. [35]	Empirical wavelet transformation with SVM	88.70	91.13	89.29
Siuly et al. [2]	EMD-based features with EBT	89.59	89.76	89.32
Guo et al. [38]	ERP features with RF	81.10	NA	NA
Khare and Bajaj [37]	F-TQWT-based scheme	91.39	92.65	93.22
Guo et al. [38]	Electrical marker with CNN	92.00	NA	NA
Khare et al. [38]	RVMD-based OELM method	92.93	97.15	91.06
Khare and Bajaj [39]	SPWVD-based TFR and CNN model	93.36	94.25	92.03
Proposed method	GoogLeNet-based deep features with an SVM model	**98.84**	**99.02**	**98.58**

^*∗*^NA = not available.

Bold values represent the highest performance.

## Data Availability

The data can be obtained from the Kaggle website: https://www.kaggle.com/broach/button-tone-sz.
